# The Relative Risk of Immune-Related Liver Dysfunction of PD-1/PD-L1 Inhibitors Versus Chemotherapy in Solid Tumors: A Meta-Analysis of Randomized Controlled Trials

**DOI:** 10.3389/fphar.2019.01063

**Published:** 2019-09-23

**Authors:** Siyao Deng, Qinyan Yang, Xiaochen Shu, Jinyi Lang, Shun Lu

**Affiliations:** ^1^School of Medicine, University of Electronic Science and Technology of China, Chengdu, China; ^2^Department of Hepatobiliary Surgery and Cell Transplantation Center, Sichuan Academy of Medical Sciences & Sichuan Provincial People’s Hospital, Chengdu, China; ^3^Department of Epidemiology, School of Public Health, Medical College of Soochow University, Suzhou, China; ^4^Department of Radiation Oncology, Sichuan Cancer Hospital & Institute, Sichuan Cancer Center, School of Medicine, University of Electronic Science and Technology of China, Chengdu, China; ^5^Department of Radiological Protection, Radiation Oncology Key Laboratory of Sichuan Province, Chengdu, China

**Keywords:** immune checkpoint inhibitors, PD-1/PD-L1 inhibitors, immune-related adverse events, liver dysfunction, Nivolumab, Pembrolizumab, Atezolizumab

## Abstract

**Background:** Immune checkpoint inhibitors (ICIs) have made a significant breakthrough in the treatment of solid tumors; however, their use also generates unique immune-related adverse effects (irAEs). Here, we performed a systematic review and meta-analysis to assess the risk of immune-related liver dysfunction between in patients treated by programmed death 1 (PD-1)/programmed death ligand 1 (PD-L1) inhibitors exclusively and chemotherapy.

**Methods:** A comprehensive search of multiple databases identified eligible studies, including randomized controlled trials (RCTs) with PD-1/PD-L1 inhibitors exclusively and chemotherapy in patients with different solid tumors was carried out. The elevations of alanine aminotransferase (ALT) and aspartic aminotransferase (AST) were used to evaluate liver dysfunction. The relative risk (RR) and 95% confidence intervals (CI) were calculated and analyzed by Review Manager 5.3 and STATA version 12.0 statistical software.

**Results:** After screening and eligibility assessment, a total of 5638 patients from 12 RCTs were included in our meta-analysis. In comparison with chemotherapy, patients treated with PD-1/PD-L1 inhibitors exclusively showed an increased incidence of all-grade ALT/AST elevations (ALT: RR, 1.52, 95% CI, 1.09–2.13; *p* = 0.01; AST: RR, 1.96, 95% CI, 1.37–2.81; *p =* 0.0002). Patients receiving PD-1 inhibitors showed the significantly higher risk of all-grade ALT/AST elevations incidence than those receiving chemotherapy (ALT: RR, 1.47; 95% CI, 1.05–2.07; *p =* 0.03; AST: RR, 1.90, 95% CI, 1.32–2.73; *p* = 0.0005). However, no significant difference was found between PD-L1 inhibitor and chemotherapy group. Moreover, for non-small cell lung cancer (NSCLC) and urothelial carcinoma (UC), patients treated with PD-1/PD-L1 inhibitors exclusively exhibited a significant higher risk of all-grade ALT elevation incidence (NSCLC: RR, 1.92; 95% CI, 1.23–3.02; *p* = 0.004; UC: RR, 3.36; 95% CI, 1.12–10.06, *p* = 0.03) and all-grade AST elevation incidence (NSCLC: RR, 2.37; 95% CI, 1.45–3.87, *p* = 0.0005; UC: RR, 4.47; 95% CI, 1.30–15.38, *p =* 0.02) than chemotherapy.

**Conclusions:** The meta-analysis confirms that PD-1/PD-L1 inhibitors exclusive pose an increased risk of immune-related liver dysfunction than chemotherapy. PD-1/PD-L1 blockade in NSCLC and UC increase the risk of immune-related liver dysfunction, but not in melanoma (MM) and head-neck squamous cell carcinoma (HNSCC).

## Introduction

Immune checkpoint blockade has become a most recent frontline of cancer treatment, since it significantly prolongs survival with fewer side effects compared with traditional chemotherapy (Gong et al., 2018). Despite the impressive antitumor immune response induced by the immune checkpoint inhibitors (ICIs), by blocking the negative immune regulatory mechanism that are normally vital for maintaining immunologic homeostasis, these agents also lead to autoimmune-like toxicities termed immune-related adverse events (irAEs) (Jing et al., 2016, Davies and Duffield, 2017). IrAEs are quite different both in mechanism and management of adverse effects induced by chemotherapy (Sznol et al., 2017), they most commonly include pruritus, diarrhea, rash, colitis, endocrine dysfunction, nephritis, liver dysfunction, and pneumonitis. Among these irAEs, immune-related liver dysfunction is usually asymptomatic and has only been discovered in routine liver function examination. Thus, it is usually ignored by clinicians. However, this liver dysfunction tends to present with higher severity and may be fatal. Explosive hepatitis with jaundice and liver failure has been reported in the treatment of Ipilimumab, highlighting the need for seriously attention (Chmiel et al., 2011). To date, clinical experience, especially the identification and therapy, has still been very scarce.

According to the permission of Food and Drug Administration (FDA), ICIs are mainly used in patients with advanced cancer or metastatic tumor. Improving the quality of life was considered as important as the prolongation of survival in these patients. Therefore, pursuing a balance between toxicity and curative effect of treatment became crucial for decision making. The side effect of traditional cytotoxicity chemotherapy was well known by plenty through clinical experience. It is urgent to compare the toxicity of ICI therapy with chemotherapy. Furthermore, with the outstanding clinical outcome of ICI treatment, the use of ICIs is expanding rapidly. It is necessary to improve our understanding about this specific side effect.

This meta-analysis was designed to determine the risk of immune-related liver dysfunction by evaluated the elevations of alanine aminotransferase (ALT) and aspartic aminotransferase (AST) in patients with solid tumors treated with programmed death 1 (PD-1)/programmed death ligand 1 (PD-L1) inhibitors exclusively or chemotherapy.

## Methods

### Search Strategy

Original articles were from the following databases: the Embase, Medline, Web of Science, and PubMed (up to December 31, 2018). Studies on the risk of immune-related liver dysfunction in PD-1/PD-L1 inhibitors therapies exclusive versus chemotherapy were searched. The following keywords and corresponding Medical Subject Heading terms were used for analyses: “ICIs,” “immune checkpoint inhibitors,” “Nivolumab,” “Pembrolizumab,” “Atezolizumab,” “PD-1 inhibitor,” “PD-L1 inhibitor,” “cancer,” “tumor,” “carcinoma,” “phase II,” and “phase III”.

### Selection and Exclusion Criteria

Studies meeting the following criteria were included in our meta-analysis: 1) phase II/III randomized controlled trials (RCTs) with primary endpoints, such as overall survival (OS), progression-free survival (PFS), or objective response rate (ORR); 2) histologically confirmed solid carcinomas; 3) random assignment of participants to treatment with single-agent PD-1/PD-L1 inhibitors or chemotherapy; 4) information of immune-related liver dysfunction for all-grade (1–5) and high-grade (3–5). Two independent reviewers screened the studies based on the key terms contained in the titles and abstracts. Then, the full texts of all potentially eligible studies were assessed. The references of relevant studies were also revised to identify other suitable studies. Letters, expert opinions, case reports, reviews, articles without available data, and duplicate publications were excluded.

### Data Extraction

Two independent investigators performed data extraction and evaluated the identified studies by using a patient, intervention, comparison, and outcome (PICO) chart (Huang et al., 2006). Discrepancies between the two reviewers were resolved by a third reviewer. The following information was recorded from the selected studies: first author’s name, year of publication, trial phase, type of solid tumors, the primary endpoint, therapeutic regimen, number of patients in the PD-1/PD-L1 inhibitors treatment or control group, number of patients enduring immune-related liver dysfunction of all-grade (1–5; recorded according to Version 4 of the Common Terminology Criteria for Adverse Events of the National Cancer Institute) and high-grade (3–5) (Basch et al., 2014).

### Statistical Analysis

The data analysis, including the comparison of the incidence and relative risk (RR) of liver dysfunction between PD-1/PD-L1 inhibitors exclusive and chemotherapy, was performed using Review Manager 5.3 (Cochrane Collaboration 2014, Nordic Cochrane Center, Copenhagen, Denmark) and STATA version 12.0 statistical software (STATA Corporation, College Station, TX, USA). The RR and the corresponding 95% confidence intervals (CIs) were calculated in patients assigned to PD-1/PD-L1 inhibitors exclusively compared with those assigned to chemotherapy in the same trial. RR >1.0 indicates a higher risk or higher incidence of liver dysfunction in patients treated with PD-1/PD-L1 inhibitors exclusively than those treated with chemotherapy. For the calculation of the RR, random or fixed-effect models were used, depending on the heterogeneity of included studies. The Q test and *I*^2^ statistics were used to assess the heterogeneity among the RCTs. When substantial heterogeneity (*p* > 0.05 or *I*^2^< 50%) was not observed, the pooled estimate was calculated based on the fixed-effect model. When substantial heterogeneity (*p* < 0.05 or *I*^2^ > 50%) was observed in the analysis, the random-effect model was used for the meta-analysis (Higgins et al., 2003, DerSimonian and Laird, 2015). Sensitivity analysis was performed by deleting one study at a time to determine if the results would be affected by a single study, particularly facing with a suspicious result or considerable heterogeneity. Subgroup analysis was conducted according to different PD-1/PD-L1 inhibitors and different types of cancer to explore the source of heterogeneity. We evaluated potential publication bias using the Begg’s and Egger’s tests with funnel plots (Begg and Mazumdar, 1994, Sterne et al., 2000). A two-tailed *p* value < 0.05 was considered statistically significant.

### Quality Assessment

To assess the risk of bias for the included studies, the Cochrane risk of bias tool was used. This tool assesses each trial for selection bias (including both random sequence generation and allocation concealment), performance bias (blinding of participants and personnel), detection bias (blinding of outcome assessment), attrition bias (incomplete outcome data), reporting bias (selective reporting), and other bias (Higgins et al., 2011). Trials with more than two and four high-risk components were considered to have a moderate and high risk of bias, respectively.

## Results

### Search Results and Study Characteristics

**Figure 1:**
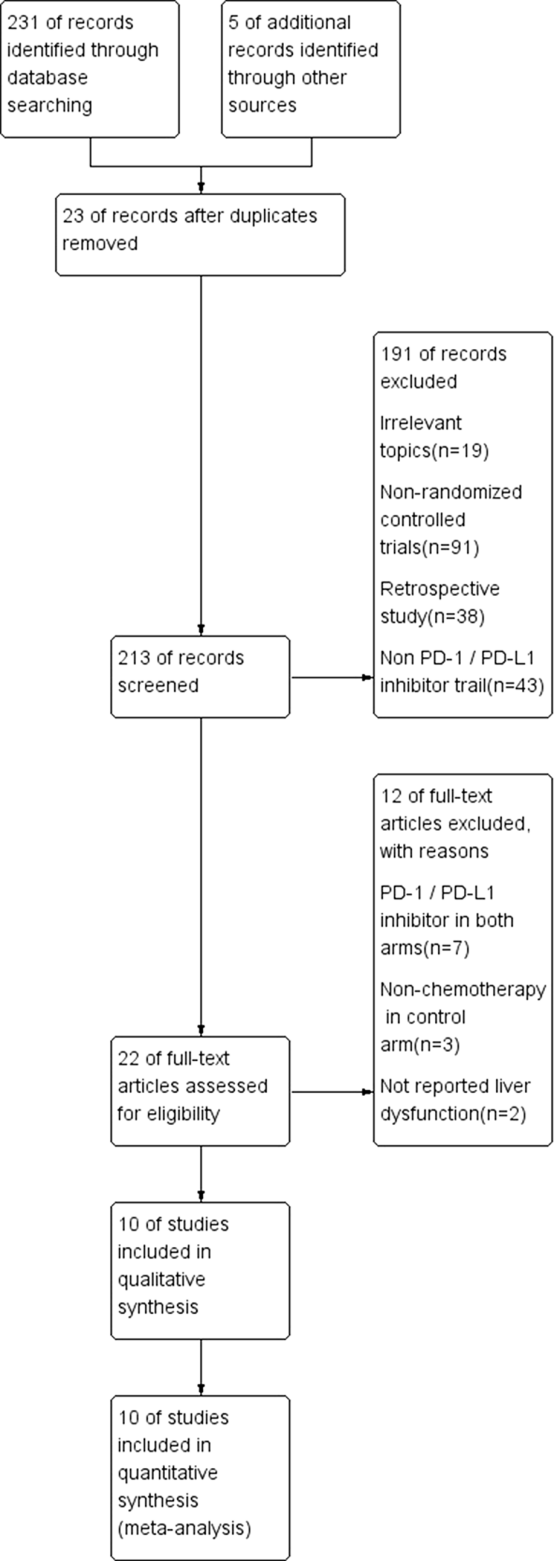
A flowchart describing the RCT selection process.

Among the 236 studies included in our database, after duplication removal, a total of 12 studies were selected (Borghaei et al., 2015, Brahmer et al., 2015, Caroline et al., 2015, Robert et al., 2015, Weber et al., 2015, Fehrenbacher et al., 2016, Ferris et al., 2016, Herbst et al., 2016, Bellmunt et al., 2017, Carbone et al., 2017). Nine of the 12 studies came from the United States and three from France. The patients enrolled in the 12 studies are all Caucasian population. Selection process and exclusion reasons are shown in **Figure 1**. A total of 5638 patients (PD-1/PD-L1 inhibitors: 3040; chemotherapy: 2598) were included in the analysis from six nivolumab trials, three pembrolizumab trials, and one atezolizumab trial. Tumor types tested in these studies included non-small cell lung cancer (NSCLC) (n = 5), melanoma (MM) (n = 3), urothelial carcinoma (UC) (n = 1), and head-neck squamous cell carcinoma (HNSCC) (n = 1). Two of the studies involved three-arm trials, in which two doses of pembrolizumab arms were compared with chemotherapy treatment. The baseline characteristics of each trial are outlined in **Table 1**.

**Table 1:** Characteristics of the eligible RCTs.

Study[year]	Country	Study type	Histology	Endpoint	Treatment arms	patients	ALT (G1-5)	ALT (G3-5)	AST (G1-5)	AST (G3-5)
Brahmer et al. (2015)	USA	RCT III	NSCLC	OS	nivolumab 3 mg/kg q2w	131	2	0	2	0
					DOX 75 mg/m^2^ q3w	129	1	1	1	1
Borghaei et al. (2015)	USA	RCT III	NSCLC	OS	nivolumab 3 mg/kg q2w	287	9	0	9	1
					DOX 75 mg/m^2^ q3w	268	4	1	2	0
Herbst1 (2016)	USA	RCT III	NSCLC	OS	pembrolizumab 2 mg/kg q2w	339	16	2	10	2
					DOX 75 mg/m^2^ q3w	309	4	0	3	0
Herbst2 (2016)	USA	RCT III	NSCLC	OS	pembrolizumab 10 mg/kg q2w	343	8	1	7	0
					DOX 75 mg/m^2^ q3w	309	4	0	3	0
Fehrenbacher et al. (2016)	USA	RCT II	NSCLC	OS	atezolizumab 1200 mg q3w	142	6	3	6	3
					DOX 75 mg/m^2^ q3w	135	1	0	1	0
Carbone et al. (2017)	USA	RCT III	NSCLC	OS	nivolumab 3 mg/kg q2w	267	19	7	23	7
					chemotherapy control	263	14	2	12	1
Weber et al. (2015)	USA	RCT III	MM	ORR	nivolumab 3 mg/kg q2w	268	7	2	11	1
					chemotherapy control	102	1	0	2	0
Robert et al. (2015)	France	RCT III	MM	OS	nivolumab 3 mg/kg q2w	206	3	2	2	1
					dacarbazine 1000 mg/m^2^ q3w	205	3	1	4	1
Schachter1 (2015)	France	RCT III	MM	OS	pembrolizumab 10 mg/kg q2w	278	12	0	14	0
					chemotherapy control	256	9	2	6	2
Schachter1 (2015)	France	RCT III	MM	OS	pembrolizumab 10 mg/kg q3w	277	4	1	6	1
					chemotherapy control	256	9	2	6	2
Bellmunt et al. (2017)	USA	RCT III	Urothelial Ca	OS PFS	pembrolizumab 200 mg q3w	266	14	3	14	6
					chemotherapy control	255	4	0	3	0
Ferris et al. (2016)	USA	RCT III	head neck	OS	nivolumab 3 mg/kg q2w	236	2	1	2	0
					chemotherapy control	111	3	1	2	0

NSCLC, non-small cell lung cancer; MM, melanoma; Urothelial Ca, urothelial carcinoma; head neck, head-neck squamous cell carcinoma. DOX, docetaxel; PFS, progression-free survival; OS, overall survival; ORR: objective response rate. Both Herbst1 and Herbst2 belong to Herbst et al 2016. And both Schachter1 and Schachter2 belong to Schachter et al 2015. Herbst1, pembrolizumab 2mg/kg q2w; Herbst2, pembrolizumab 10mg/kg q2w; Schachter1, pembrolizumab10mg/kg q2w; Schachter2, pembrolizumab 10mg/kg q3w.

**Figure 2:**
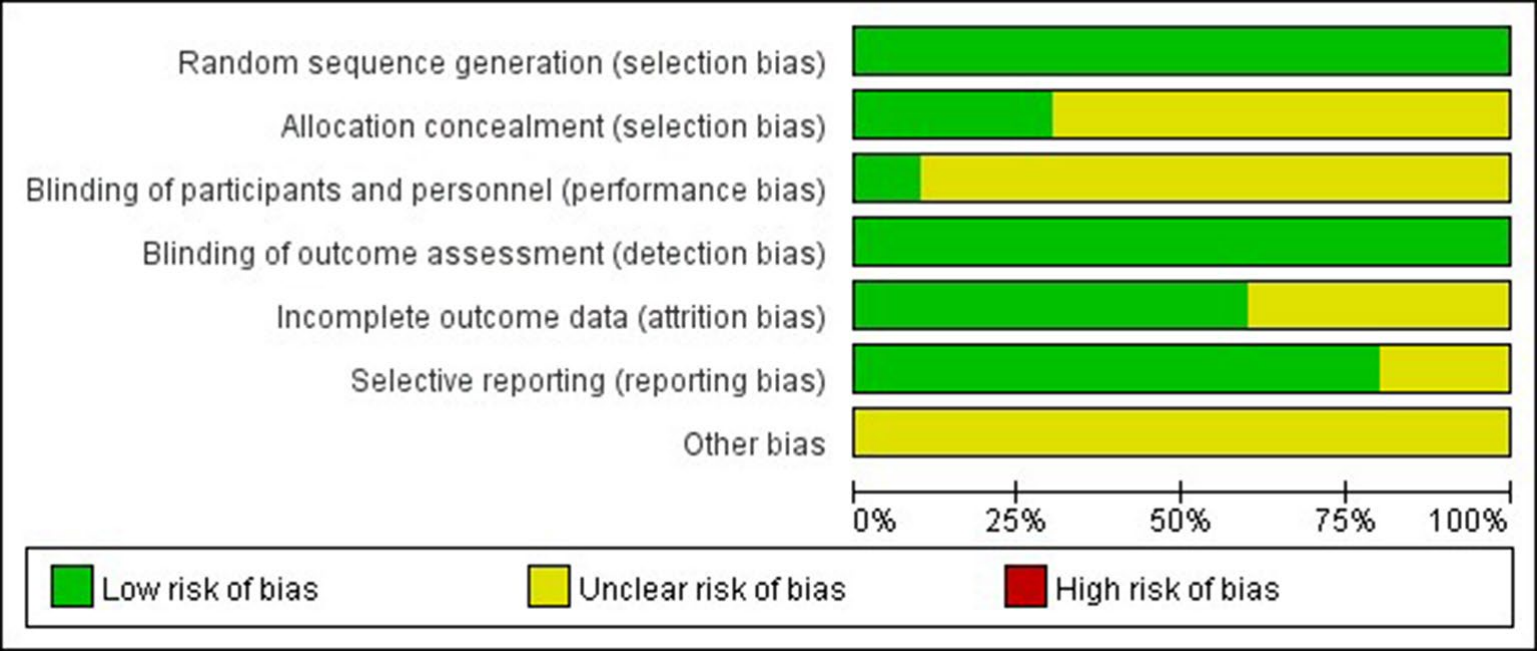
Risk of bias summary. Bar chart comparing the percentage risk of bias for each included RCT. Low risk of bias (green), high risk of bias (red), and unclear risk of bias (yellow).

**Figure 3:**
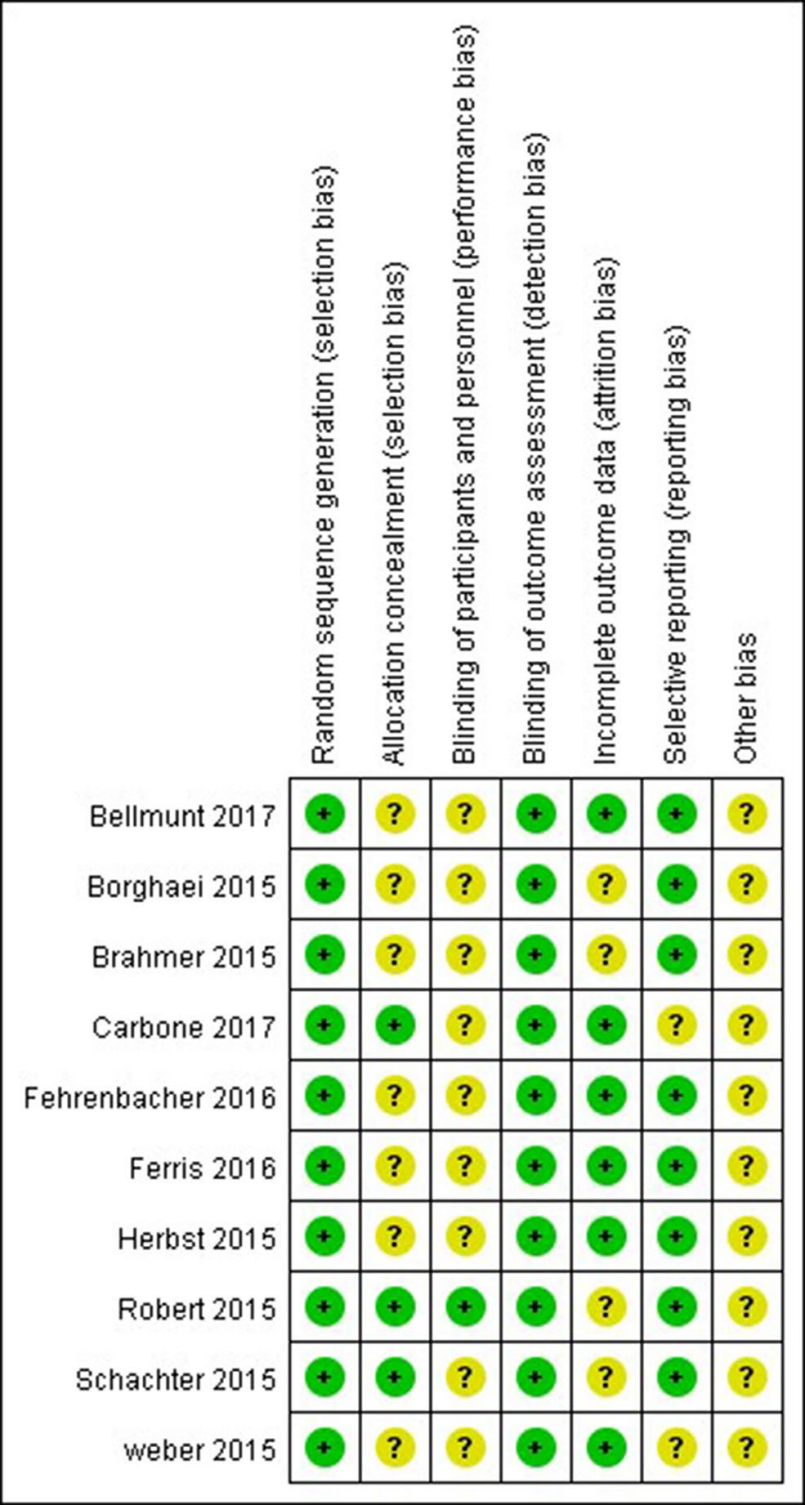
Risk of bias summary. Risk of bias for each included RCT, representing low risk of bias (+), high risk of bias (−), and unclear risk of bias (?).

The Cochrane risk of bias tool was used to evaluate the quality of each study. As shown in **Figures 2**, 3 the overall risk of bias was assessed as low risk, and all included studies were qualified.

### RR of ALT and AST Elevations Incidence by Treating With PD-1/PD-L1 Inhibitors or Chemotherapy

**Figure 4:**
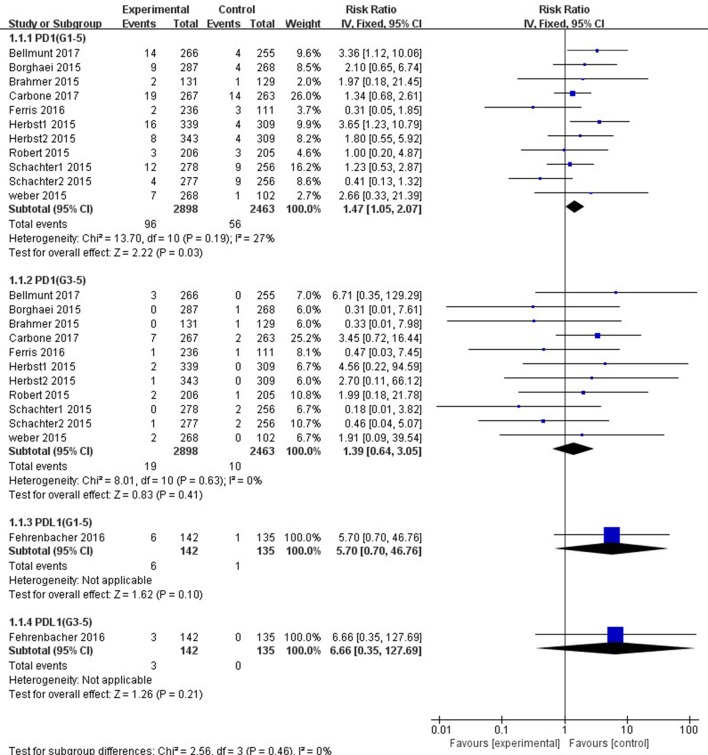
Forest plot analysis for ALT elevation with PD-1/PD-L1 inhibitors versus chemotherapy. G1-5: grades 1–5; G3-5: grades 3–5.

**Figure 5:**
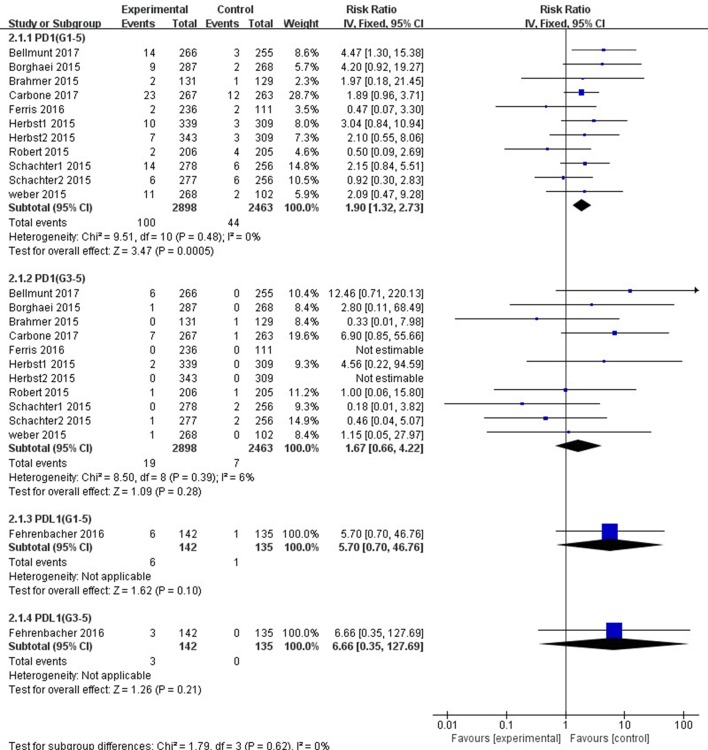
Forest plot analysis for AST elevation with PD-1/PD-L1 inhibitors versus chemotherapy. G1-5: grades 1–5; G3-5: grades 3–5.

Patients treated with PD-1 inhibitor showed a significantly higher risk of all-grade ALT and AST elevations incidence than those treated with chemotherapy (ALT: RR, 1.47; 95% CI, 1.05–2.07; *p =* 0.03; AST: RR, 1.90; 95% CI, 1.32–2.73; *p =* 0.0005, respectively) (**Figures 4**, 5). However, no significant difference in the risk of all-grade ALT or AST elevations incidence was found between PD-L1 inhibitor (atezolizumab) and chemotherapy (ALT: RR, 5.70; 95% CI, 0.70–46.76; *p* = 0.10; AST: RR, 5.70; 95% CI, 0.70–46.76; *p* = 0.10, respectively). Moreover, there was neither significant difference in the pooled RR of high-grade ALT elevation (PD-1 inhibitor: RR, 1.39; 95% CI, 0.64–3.05; *p* = 0.41; PD-L1 inhibitor: RR, 6.66; 95% CI, 0.35–127.69; *p* = 0.21) nor AST elevation (PD-1 inhibitor: RR, 1.67; 95% CI, 0.66–4.22; *p =* 0.28; PD-L1 inhibitor: RR, 6.66; 95% CI, 0.35–127.69; *p =* 0.21) between patients treated with PD-1/PD-L1 inhibitors and chemotherapy.

### Subgroup Analysis of ALT and AST Elevations Incidence by Drug

**Figure 6:**
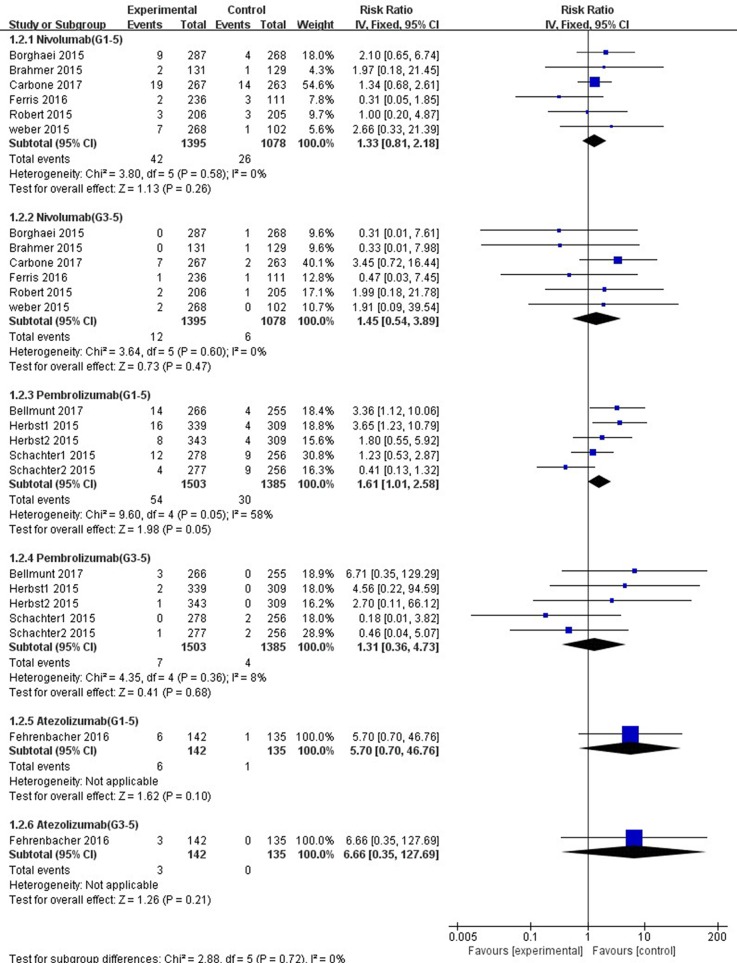
Forest plot analysis for ALT elevation with different type of immune checkpoint inhibitors (nivolumab, pembrolizumab, and atezolizumab) versus chemotherapy. G1-5: grades 1–5; G3-5: grades 3–5.

**Figure 7:**
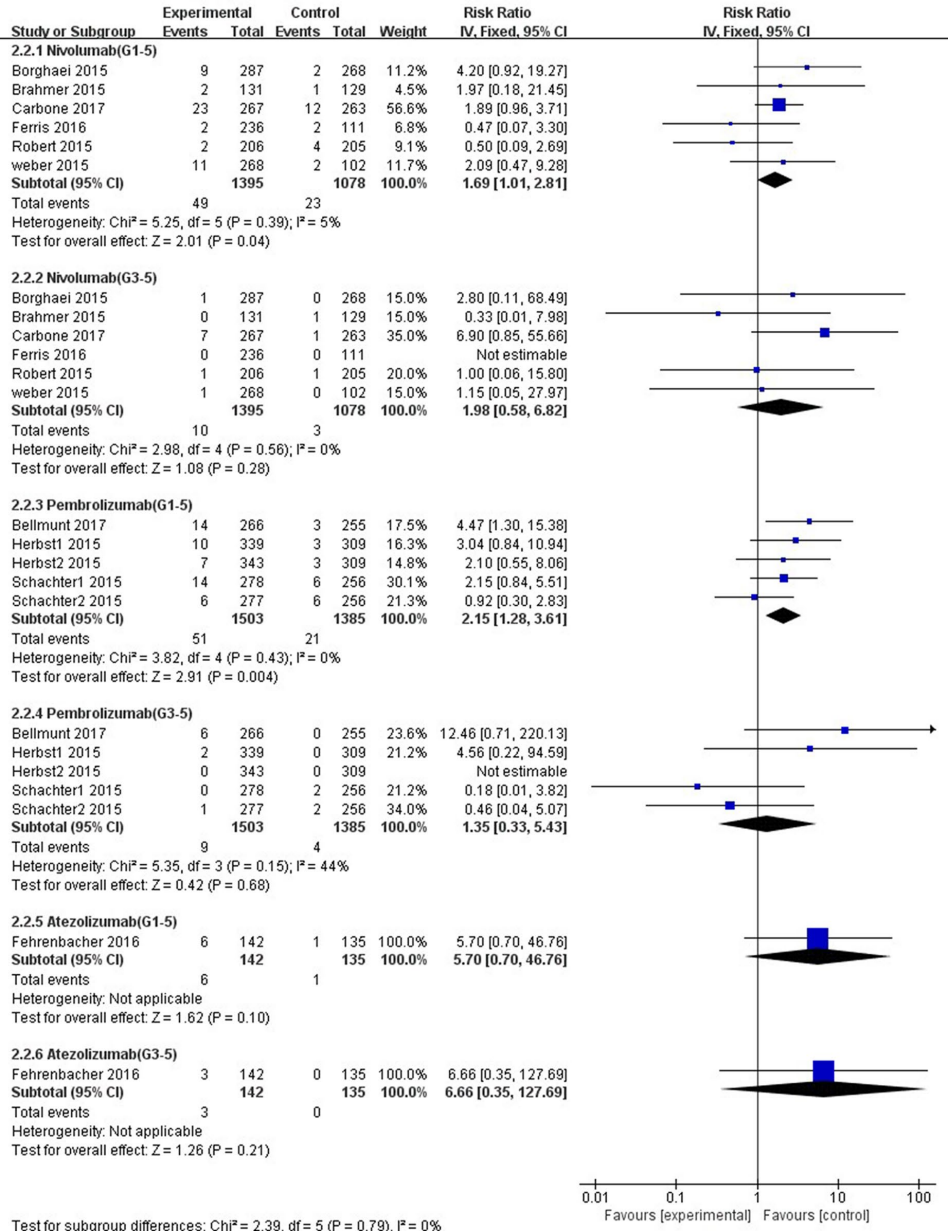
Forest plot analysis for AST elevation with different type of immune checkpoint inhibitors (nivolumab, pembrolizumab, and atezolizumab) versus chemotherapy. G1-5: grades 1–5; G3-5: grades 3–5.

In comparison with chemotherapy, patients receiving pembrolizumab achieved a significantly higher risk of all-grade ALT and AST elevations incidence (ALT: RR, 1.61; 95% CI, 1.01–2.58; *p* = 0.05; AST: RR, 2.15; 95% CI, 1.28–3.61; *p* = 0.004, respectively) (**Figures 6**, 7), but only the risk of all-grade AST elevation incidence was significantly increased in nivolumab subgroup (RR, 1.69; 95% CI, 1.01–2.81; *p =* 0.04). Furthermore, we found no significant differences between nivolumab or pembrolizumab and chemotherapy in pooled RR of high-grade ALT elevation (nivolumab: RR, 1.45; 95% CI, 0.54–3.89; *p* = 0.47; pembrolizumab: RR, 1.31; 95% CI, 0.36–4.73; *p =* 0.68) and AST elevation (nivolumab: RR, 1.98; 95% CI, 0.58–6.82; *p* = 0.28; pembrolizumab: RR, 1.35, 95% CI, 0.33–5.43; *p* = 0.68).

The overall heterogeneity of incidence for all-grade ALT elevation was tiny in the nivolumab subgroup, low in the PD-1 inhibitor subgroup, and moderate in the pembrolizumab subgroup (nivolumab: *I*^2^ = 0%, *p* = 0.58; PD-1: *I*^2^ = 27%, *p =* 0.19; pembrolizumab: *I*^2^ = 58%, *p* = 0.05, respectively). Additionally, regarding high-grade ALT and all-grade AST elevation incidence, a small heterogeneity was observed in the nivolumab subgroup (ALT: *I*^2^ = 0%, *p* = 0.60; AST: *I*^2^ = 5%, *p* = 0.39, respectively), the pembrolizumab subgroup (ALT: *I*^2^ = 8%, *p* = 0.36; AST: *I*^2^ = 0%, *p* = 0.43, respectively), and the PD-1 inhibitor subgroup (ALT: *I*^2^ = 0%, *p* = 0.63; AST: *I*^2^ = 0%, *p* = 0.48, respectively). Of note, for high-grade AST elevation incidence, we not only found a small heterogeneity in the PD-1 inhibitor subgroup (*I*^2^ = 6%, *p =* 0.39) and the nivolumab subgroup (*I*^2^ = 0%, *p* = 0.56) but also a moderate heterogeneity in the pembrolizumab subgroup (*I*^2^ = 44%, *p =* 0.15). The fixed-effect model was used for the RR analysis of all- and high-grade ALT and AST elevations incidence, due to an overall lack of heterogeneity within the included studies.

As shown in **Tables 2**, **3**, **4**, the sensitivity analysis was performed to detect whether the results could have an impact on the PD-1 (grades 1–5 ALT elevation) subgroup (*I*^2^ = 27%), the pembrolizumab (grades 1–5 ALT elevation) subgroup (*I*^2^ = 58%), and the pembrolizumab (grades 3–5 AST elevation) subgroup (*I*^2^ = 44%), respectively.

**Table 2:** Sensitivity analysis for ALT elevation (Grade1-5) in patients treated with PD-1 inhibitor versus chemotherapy.

Removed study	Trails	Heterogeneity		RR (95% CI)	P
		P	I^2^		
All Study	11	0.19	27%	1.47 (1.05–2.07)	0.03
Bellmunt et al. (2017)	10	0.26	20%	1.35 (0.94–1.93)	0.10
Borghaei et al. (2015)	10	0.15	32%	1.42 (1.04–2.03)	0.05
Brahmer et al. (2015)	10	0.14	34%	1.46 (1.04–2.06)	0.03
Carbone et al. (2017)	10	0.14	34%	1.52 (1.02–2.26)	0.04
Ferris et al. (2016)	10	0.30	16%	1.56 (1.10–2.26)	0.01
Herbst1 (2016)	10	0.30	16%	1.33 (0.93–1.91)	0.12
Herbst2 (2016)	10	0.14	34%	1.44 (1.01–2.06)	0.04
Robert et al. (2015)	10	0.14	34%	1.50 (1.06–2.12)	0.02
Schachter1 (2015)	10	0.14	33%	1.52 (1.05–2.21)	0.03
Schachter2 (2015)	**10**	**0.47**	**0%**	**1.66 (1.16–2.37)**	**0.005**
Weber et al. (2015)	10	0.15	33%	1.45 (1.02–2.04)	0.04

The bold text indicates that this study is the main source of heterogeneity in the subgroup.

**Table 3:** Sensitivity analysis for ALT elevation (Grade1-5) in patients treated with pembrolizumab versus chemotherapy.

Removed study	Trails	Heterogeneity		RR (95% CI)	P
		P	I^2^		
All Study	5	0.05	58%	1.61 (1.01–2.58)	0.05
Bellmunt et al. (2017)	4	0.06	60%	1.36 (0.81–2.30)	0.24
Herbst1 (2016)	4	0.07	57%	1.33 (0.79–2.25)	0.28
Herbst2 (2016)	4	0.02	69%	1.58 (0.94–2.63)	0.08
Schachter1 (2015)	4	0.04	67%	1.82 (1.03–3.20)	0.04
Schachter2 (2015)	**4**	**0.35**	**9%**	**2.10 (1.26–3.51)**	**0.005**

The bold text indicates that this study is the main source of heterogeneity in the subgroup.

**Table 4:** Sensitivity analysis for AST elevation (Grade3-5) in patients treated with pembrolizumab versus chemotherapy.

Removed study	Trails	Heterogeneity		RR (95% CI)	P
		P	I^2^		
All Study	5	0.15	44%	1.35 (0.33–5.43)	0.68
Bellmunt et al. (2017)	**4**	**0.31**	**14%**	**0.68 (0.14–3.34)**	**0.63**
Herbst1 (2016)	4	0.10	56%	0.97 (0.20–4.67)	0.97
Herbst2 (2016)	4	0.15	44%	1.35 (0.33–5.43)	0.68
Schachter1 (2015)	4	0.20	38%	2.30 (0.48–11.07)	0.30
Schachter2 (2015)	4	0.12	52%	2.33 (0.42–13.00)	0.33

The bold text indicates that this study is the main source of heterogeneity in the subgroup.

### Subgroup Analysis of ALT and AST Elevations Incidence by Cancer Type

**Figure 8:**
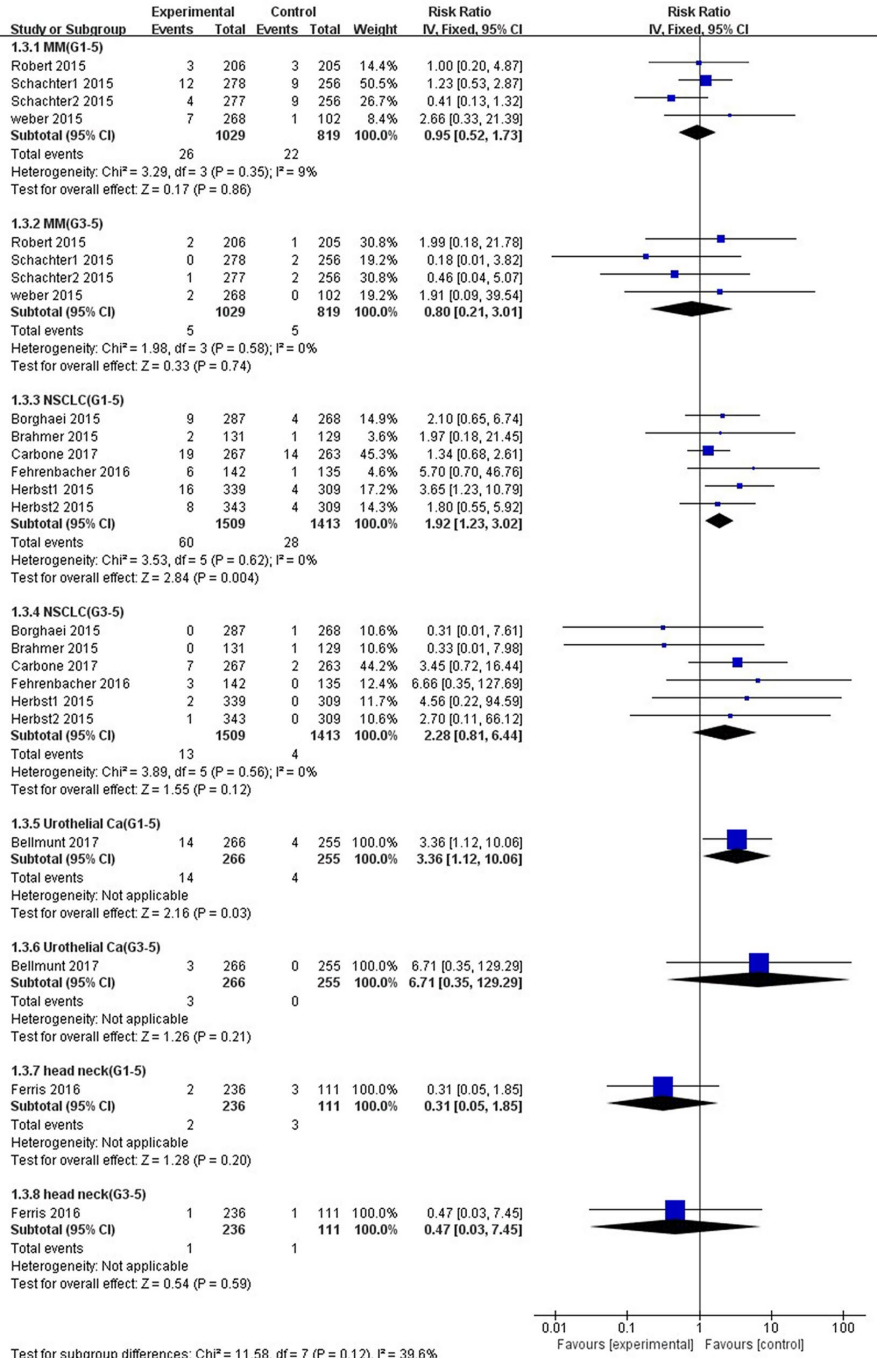
Forest plot analysis for ALT elevation in different cancers with PD-1/PD-L1 inhibitors versus chemotherapy. MM: melanoma; NSCLC: non-small cell lung cancer; Urothelial Ca: urothelial carcinoma; head neck: head-neck squamous cell carcinoma. G1-5: grade 1–5; G3-5: grade 3–5.

As shown in **Figure 8**, the risk of all-grade ALT elevation incidence significantly increased in patients with NSCLC and UC treated by PD-1/PD-L1 inhibitors than chemotherapy (NSCLC: RR, 1.92; 95% CI, 1.23–3.02; *p* = 0.004; UC: RR, 3.36; 95% CI, 1.12–10.06; *p* = 0.03), but did not change significantly in patients with MM and HNSCC (MM: RR, 0.95; 95% CI, 0.52–1.73; *p* = 0.86; HNSCC: RR, 0.31; 95% CI, 0.05–1.85; *p* = 0.20). Additionally, with respect to high-grade ALT elevation, treatment with PD-1/PD-L1 inhibitors did not significantly increase the pooled RR of ALT elevation incidence in patients suffering from NSCLC (RR, 2.28; 95% CI, 0.81–6.44; *p* = 0.12) and UC (RR, 6.71; 95% CI, 0.35–129.29; *p* = 0.21).

**Figure 9:**
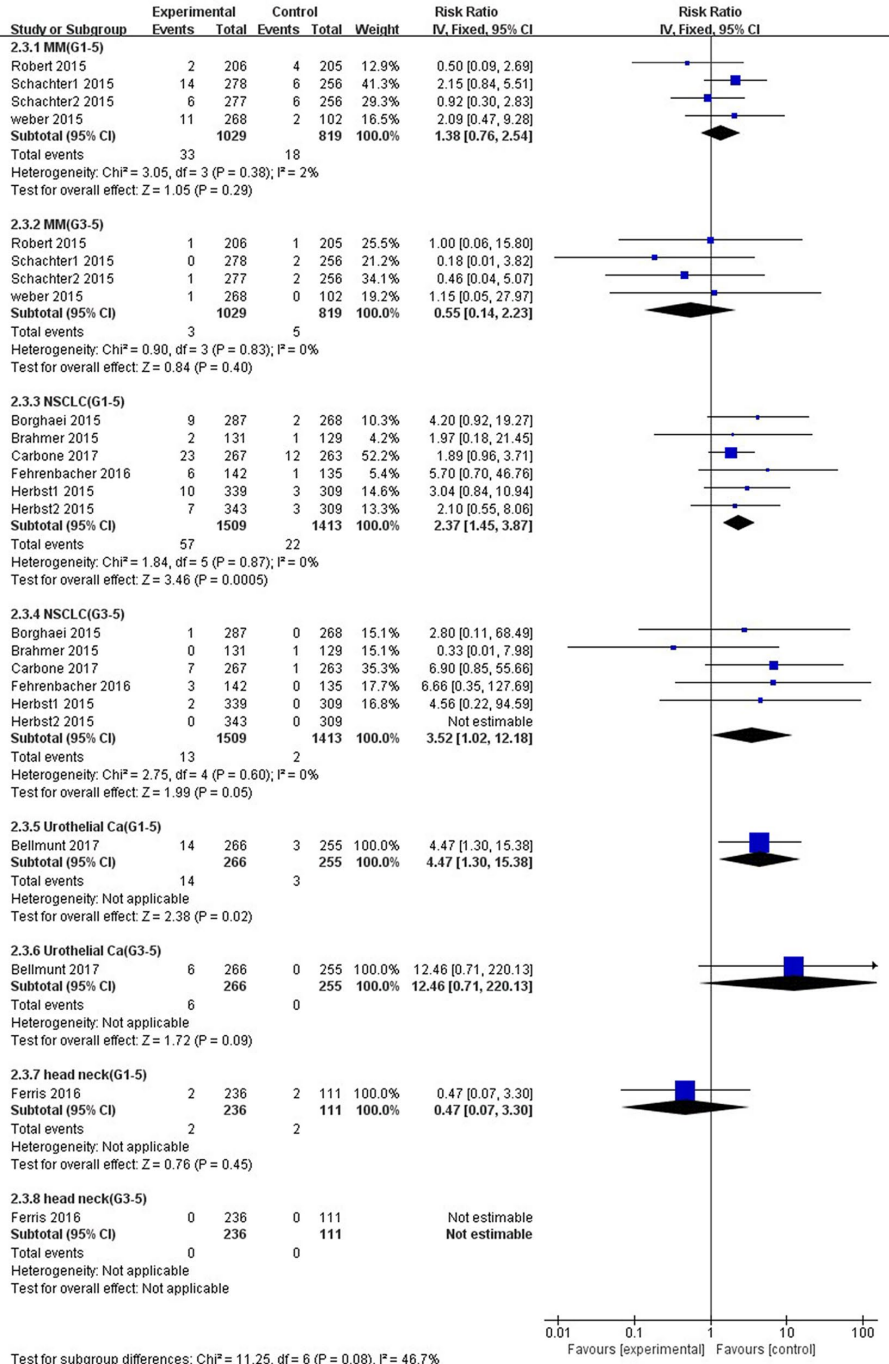
Forest plot analysis for AST elevation in different cancers with PD-1/PD-L1 inhibitors versus chemotherapy. MM: melanoma; NSCLC: non-small cell lung cancer; Urothelial Ca: urothelial carcinoma; head neck: head-neck squamous cell carcinoma. G1-5: grade 1–5; G3-5: grades 3–5.

Compared with chemotherapy, significant higher risk of all-grade AST elevation incidence was observed in patients with NSCLC (RR 2.37, 95% CI, 1.45–3.87, *p* = 0.0005) and UC (RR 4.47, 95% CI, 1.30–15.38, *p* = 0.02) treated with PD-1/PD-L1 inhibitors exclusively (**Figure 9**). However, no significant difference of all-grade AST elevation incidence was found in patients with either MM (RR, 1.38; 95% CI, 0.76–2.54; *p* = 0.29) or HNSCC (RR, 0.47; 95% CI, 0.07–3.30; *p* = 0.45). Furthermore, in regard to high-grade AST elevation, NSCLC patients treated with PD-1/PD-L1 inhibitors showed a significantly higher RR of AST elevation incidence (RR, 3.52; 95% CI, 1.02–12.18; *p =* 0.05) than those treated with chemotherapy, but this difference was not observed in UC patients (RR, 12.46; 95% CI, 0.71–220.13; *p =* 0.09).

A small overall heterogeneity of all-grade ALT and AST elevations incidence was found in both the MM subgroup (ALT: *I*^2^ = 9%, *p* = 0.35; AST: *I*^2^ = 2%, *p* = 0.38, respectively) and the NSCLC subgroup (ALT: *I*^2^ = 0%, *p* = 0.62; AST: *I*^2^ = 0%, *p* = 0.87, respectively). As to high-grade ALT and AST elevations incidence, we also observed a tiny heterogeneity in the MM subgroup (ALT: I^2^ = 0%, *p* = 0.58; AST: *I*^2^ = 0%, *p =* 0.83, respectively) and the NSCLC subgroup (ALT: *I*^2^ = 0%, *p* = 0.56; AST: *I*^2^ = 0%, *p* = 0.60, respectively).

### Analysis of Publication Bias

**Figure 10:**
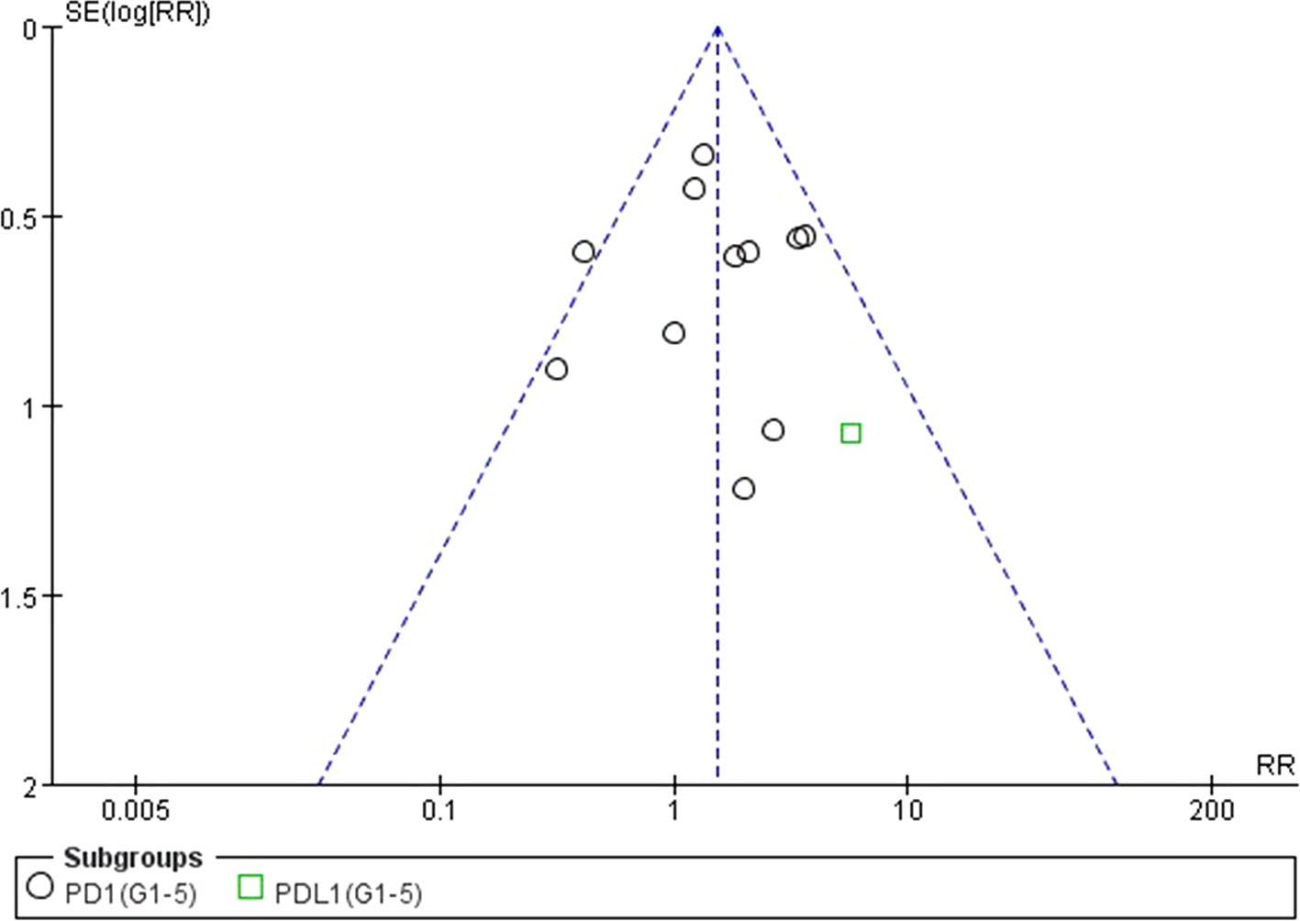
Funnel plot for ALT elevation (grades 1–5) in patients treated with PD-1 antibodies versus chemotherapy therapy.

**Figure 11:**
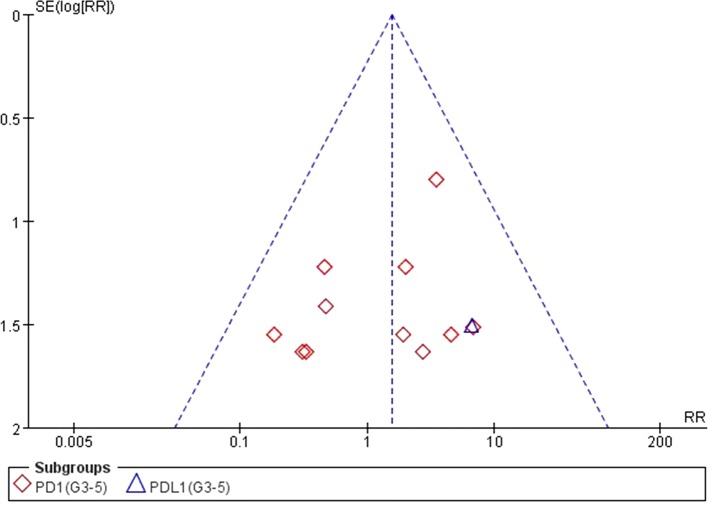
Funnel plot for ALT elevation (grades 3–5) in patients treated with PD-1 antibodies versus chemotherapy therapy.

**Figure 12:**
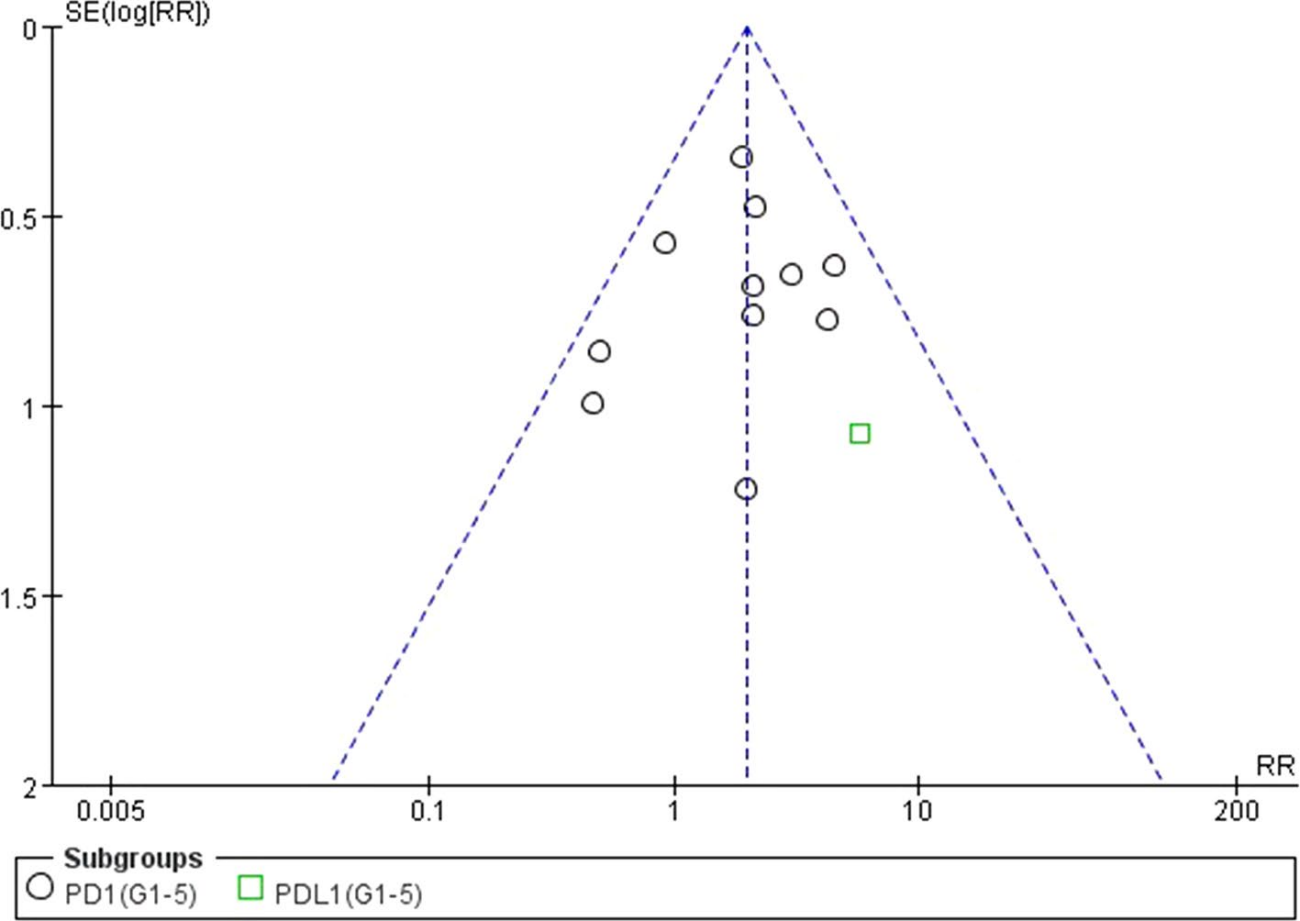
Funnel plot for AST elevation (grades 1–5) in patients treated with PD-1 antibodies versus chemotherapy therapy.

**Figure 13:**
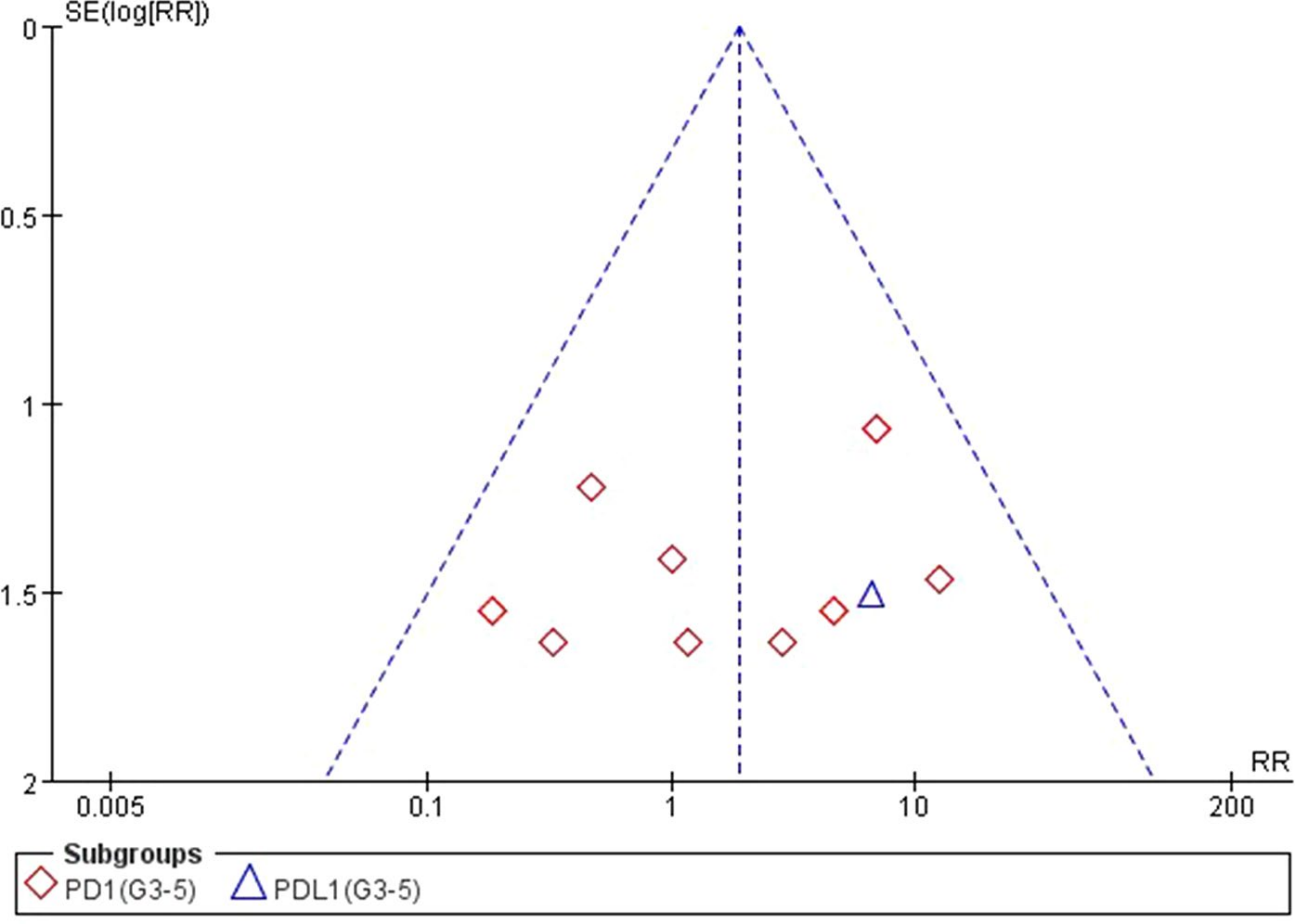
Funnel plot for AST elevation (grades 3–5) in patients treated with PD-1 antibodies versus chemotherapy therapy.

We used Egger’s test and Begg’s test conducted in STATA 12.0 software to assess the publication bias of the included literatures. As shown in **Table 5**, all the *p* values were > 0.05 after two tests. In addition, the funnel plots for a relative risk of all- and high-grade ALT/AST elevations showed that each trail was arranged symmetrically on either side of the funnel (**Figures 10**–**13**). Collectively, there was no significant publication bias in our meta-analysis.

**Table 5:** Evaluation of publication bias with Begg’s and Egger’s tests.

	Trails	Heterogeneity		RR (95% CI)	Begg’s test		Egger’s test	
		P	I^2^		Z	P	T	P
ALT elevations (G1-5)	12	0.17	28%	1.52 (1.09–2.13) p = 0.01	0.07	0.945	0.28	0.785
ALT elevations (G3-5)	12	0.62	0%	1.54 (0.72–3.29) p = 0.26	0.89	0.373	−1.09	0.301
AST elevations (G1-5)	12	0.48	0%	1.96 (1.37–2.81) p = 0.0002	0.21	0.837	−0.11	0.912
AST elevations (G3-5)	12	0.41	3%	1.89 (0.78–4.57) p = 0.16	0.36	0.721	−0.73	0.486

## Discussion

Currently, ICIs have gathered a great deal of attention as a novel promising antitumor therapy, with PD-1 or PD-L1 inhibitor demonstrating remarkable antitumor immune responses, overturning tumor-induced immune tolerance and improving survival rate of patients with malignant tumors after surgery, radiotherapy, or chemotherapy (Hodi et al., 2010, De et al., 2017). PD-1/PD-L1 inhibitors, such as nivolumab, pembrolizumab, and atezolizumab, have been approved by Food and Drug Administration (FDA) for the treatment of various advanced solid tumors, including NSCLC, UC, MM, and HNSCC; new indications are expected to rise further. With the increasing application of these agents, more and more irAEs were observed in clinic practice (Davies and Duffield, 2017, Wang et al., 2019). Among these irAEs, immune-related liver dysfunction is very insidious and usually discovered by elevations of ALT and AST in routine liver function tests instead of by clinical symptoms. It is worth noting that this autoimmune-mediated disorder may progress and even be life-threatening (Chmiel et al., 2011). Currently, both ICIs and chemotherapy are approved treatment for advanced cancer. Although for advanced cancer treatment, a lot of times, reducing the toxicity was considered as important as prolongation of survival, especially for palliative treatment in some very late stage cancer. Therefore, determining the liver dysfunction in patients with solid tumors treated with PD-1/PD-L1 inhibitors exclusively or chemotherapy is needed for informed treatment decisions.

Previous studies have demonstrated that chemotherapy has long been related to serious adverse events, whereas PD-1/PD-L1 inhibitors are generally safer than chemotherapy in most toxic events for patients (Khan et al., 2018, Luo et al., 2018). However, some recent studies have suggested that patients treated with PD-1/PD-L1 inhibitors exclusively have a higher risk of increasing the incidence of certain irAEs, such as pneumonia, colitis, and hyperthyroidism, in comparison with chemotherapy (O’Kane et al., 2017, Ma et al., 2018, Su et al., 2018). In present study, our results confirmed that patients receiving PD-1 inhibitor exclusively increased the risk of both all-grade ALT and AST elevations incidence than chemotherapy. In comparison with previous investigations, our result is more convinced with a larger number of recruited clinical trials. In addition, our study showed that there was no high-grade ALT or AST elevation found in patients treated with PD-1/PD-L1 inhibitors exclusively than chemotherapy, which provided more details of toxicity of ICIs to clinician for making treatment selection. Taken together, our finding suggested that more attention needs to be paid on advanced cancer patients with liver dysfunction, when considering treating by ICIs.

A newly published meta-analysis has demonstrated that patients treated with PD-1 inhibitor were more likely to have a higher mean incidence of grade 3 or higher adverse events than treated with PD-L1 inhibitor (Wang et al., 2019). Interestingly, our study found similar results; PD-1 inhibitor was associated with increased ALT and AST elevations incidence compared with PD-L1 inhibitor. PD-1 is known to have two ligands, PD-L1 (B7-H1/CD274) and PD-L2 (B7-DC/CD273) (Zak et al., 2017), whereas PD-L1 inhibitor only blocks binding to PD-1 (Philips and Atkins, 2015). Therefore, PD-1 inhibitor may block more level of checkpoint signaling than PD-L1 inhibitor (Friedman et al., 2016, Postow et al., 2018). It is noticeable, without well-designed clinical trials to compare the adverse events between PD-1 inhibitor and PD-L1 inhibitor, interpretation of these results need to be made carefully. On the other hand, our meta-analysis also revealed that although both nivolumab and pembrolizumab belong to PD-1 inhibitor, pembrolizumab caused more risk of ALT and AST elevations incidence when compared with nivolumab. It has been shown that nivolumab and pembrolizumab have no overlapping binding regions on the PD-1 protein (Tan et al., 2017), suggesting that the mechanism of action may be different in these two inhibitors. These differences in PD-1 binding sites between nivolumab and pembrolizumab may account for the different risk of ALT and AST elevations incidence. In this regard, our research may provide a basis for clinicians to recommend proper medications for patients.

Previous study indicated that the incidence of irAEs was different in patients with different solid tumors (Wang et al., 2019). Similarly, our results showed that the risk of ALT and AST elevations incidence was significantly higher in patients with NSCLC compared to patients with other tumors. To date, the mechanism by which this result occurs has not been well illustrated. Several studies have reported a high expression of PD-1 in NSCLC. It is also confirmed that the expression of PD-1 was related to the negative regulation of anti-tumor immune response in NSCLC (Konishi et al., 2004, Ji et al., 2016). In addition, the FDA has approved pembrolizumab as a first-line treatment for NSCLC with high PD-1 expression (> 50%) (Reck et al., 2016). Our meta-analysis suggested it may be that NSCLC cells up-regulated more PD-1, therefore, when PD-1/PD-L1 inhibitors block the binding of these receptors to their ligands, the inhibitory signals are strongly eliminated and the host’s anti-tumor response is more likely to be effectively enhanced (Rizvi et al., 2015). At the same time, normal liver tissue cells also suffer more attacks, resulting in an increased risk of ALT and AST elevations incidence in patients with NSCLC. Another reason may be that the chemotherapy regimens and doses of NSCLC are different from those of other tumor types (NSCLC: Docetaxel 75 mg/m² every 3 weeks; MM: dacarbazine 1000 mg/m² every 3 weeks; HNSCC: methotrexate or docetaxel; UC: paclitaxel, docetaxel, or vinflunine), which may cause differences in the overall original data and final results of this meta-analysis. Therefore, our results suggested that the risk of immune-related liver dysfunction incidence depends on the cancer type, and it provided the evidence for clinicians to make the appropriate treatment selection for patients with advanced cancer.

In general, relatively small heterogeneity was observed in our meta-analysis. It is logical, given that the diagnosis of ALT and AST elevations is established on the basis of liver function examination, thus, there are no subjective factors to influence the results. Our meta-analysis based on published data itself inevitably has some limitations. First, the results described in this meta-analysis are subject to the limitations of the selected individual clinical trials, this study is influenced by all the biases and errors of the original investigators. Second, given that the diagnostic criteria of ALT/AST elevations were identical for all recruited trials, the liver dysfunction may occur on account of not only drug-induced liver injury but also cancer itself, it is hard to avoid the bias of individual selection. Lastly, there are some questions that remained unclear, such as those for the two PD-1 inhibitors, nivolumab and pembrolizumab. Our results showed that only pembrolizumab caused more risk of ALT elevation than chemotherapy.

Overall, although ICIs have made great breakthroughs in the treatment of multiple types of tumors, our meta-analysis indicated that ICIs could significantly increase the risk of liver dysfunction when compared with traditional chemotherapy, especially in the NSCLC patients treated with pembrolizumab. This suggests that clinicians need to pay more attention to avoid this risk and focus on the guidelines and expert consensus on management protocols for this rare but potentially serious liver dysfunction (Haanen et al., 2017, Puzanov et al., 2017, Brahmer et al., 2018).

## Conclusion

To sum up, PD-1 inhibitor posed an increased risk of immune-related liver dysfunction compared with chemotherapy. In PD-1 inhibitor, our meta-analysis concluded that pembrolizumab is more likely to cause an increased risk of immune-related liver dysfunction than nivolumab. Moreover, the risk of immune-related liver dysfunction in NSCLC is higher than in other tumor types with the treatment of PD1/PD-L1 inhibitors. Immune-related liver dysfunction, although relatively rare in irAEs, still requires clinicians to pay closely attention, and timely formulate corresponding prevention and response strategies, as well as appropriate management measures. Although ensuring the medication is more reasonable and effective, it is necessary to further reduce the possible liver dysfunction. We expect that further research on the molecular mechanisms of immune-related liver dysfunction will provide help to prevent and mitigate this adverse event for patients with advanced cancer.

## Data Availability

All datasets generated for this study are included in the manuscript/supplementary files.

## Author Contributions

Conception and design: SD and SL. Provision of study material or patients: XS and JL. Collection and/or assembly of data: SD and QY. Data analysis and interpretation, Manuscript writing, final approval of manuscript, and equally accountable for all aspects of the work: a authors.

## Funding

Granting agency: Sichuan Science and Technology Department. Grant numbers: 2017HH0096 and 2019YFH0073

## Conflict of Interest Statement

The authors declare that the research was conducted in the absence of any commercial or financial relationships that could be construed as a potential conflict of interest.

## References

[B1] Basch E. Reeve B. B. Mitchell S. A. Clauser S. B. Minasian L. M. Dueck A. C. (2014). Development of the National Cancer Institute’s patient-reported outcomes version of the common terminology criteria for adverse events (PRO-CTCAE). J. Natl. Cancer Inst. 106 (9), dju244. 10.1093/jnci/dju244 PMC4200059 25265940

[B2] Begg C. B. Mazumdar M. (1994). Operating characteristics of a rank correlation test for publication bias. Biometrics 50 (4), 1088–1101. 10.2307/2533446 7786990

[B3] Bellmunt J. de Wit R. Vaughn D. J. Fradet Y. Lee J. L. Fong L. (2017). Pembrolizumab as Second-Line Therapy for Advanced Urothelial Carcinoma. N. Engl. J. Med. 376 (11), 1015–1026. 10.1056/NEJMoa1613683 28212060 PMC5635424

[B4] Borghaei H. Paz-Ares L. Horn L. Spigel D. R. Steins M. Ready N. E. (2015). Nivolumab versus docetaxel in advanced nonsquamous non-small-cell lung cancer. N. Engl. J. Med. 373 (17), 1627–1639. 10.1056/NEJMoa1507643 26412456 PMC5705936

[B5] Brahmer J. Reckamp K. L. Baas P. Crino L. Eberhardt W. E. Poddubskaya E. (2015). Nivolumab versus docetaxel in advanced squamous-cell non-small-cell lung cancer. N. Engl. J. Med. 373 (2), 123–135. 10.1056/NEJMoa1504627 26028407 PMC4681400

[B6] Brahmer J. R. Lacchetti C. Schneider B. J. Atkins M. B. Brassil K. J. Caterino J. M. (2018). Management of immune-related adverse events in patients treated with immune checkpoint inhibitor therapy: American Society of Clinical Oncology Clinical Practice Guideline. J. Clin. Oncol. 36 (17), 1714–1768. 10.1200/JCO.2017.77.6385 29442540 PMC6481621

[B7] Carbone D. P. Reck M. Paz-Ares L. Creelan B. Horn L. Steins M. (2017). First-line nivolumab in stage IV or recurrent non-small-cell lung cancer. N. Engl. J. Med. 376 (25), 2415–2426. 10.1056/NEJMoa1613493 28636851 PMC6487310

[B8] Caroline R. Long G. V. Benjamin B. Caroline D. Michele M. Laurent M. (2015). Nivolumab in previously untreated melanoma without BRAF mutation. N. Engl. J. Med. 372 (4), 320–330. 10.1056/NEJMoa1412082 25399552

[B9] Chmiel K. D. Suan D. Liddle C. Nankivell B. Ibrahim R. Bautista C. (2011). Resolution of severe ipilimumab-induced hepatitis after antithymocyte globulin therapy. J. Clin. Oncol. 29 (9), e237–e240. 10.1200/JCO.2010.32.2206 21220617

[B10] Davies M. Duffield E. A. (2017). Safety of checkpoint inhibitors for cancer treatment: strategies for patient monitoring and management of immune-mediated adverse events. Immunotargets Ther. 6, 51–71. 10.2147/ITT.S141577 28894725 PMC5584920

[B11] De V. G. Je Y. Bossé D. Awad M. M. Ott P. A. Moreira R. B. (2017). Comprehensive meta-analysis of key immune-related adverse events from CTLA-4 and PD-1/PD-L1 inhibitors in cancer patients. Cancer Immunol. Res. 5 (4), 312. 10.1158/2326-6066.CIR-16-0237 28246107 PMC5418853

[B12] DerSimonian R. Laird N. (2015). Meta-analysis in clinical trials revisited. Contemp. Clin. Trials 45 (Pt A), 139–145. 10.1016/j.cct.2015.09.002 26343745 PMC4639420

[B13] Fehrenbacher L. Spira A. Ballinger M. Kowanetz M. Vansteenkiste J. Mazieres J. (2016). Atezolizumab versus docetaxel for patients with previously treated non-small-cell lung cancer (POPLAR): a multicentre, open-label, phase 2 randomised controlled trial. Lancet 387 (10030), 1837–1846. 10.1016/S0140-6736(16)00587-0 26970723

[B14] Ferris R. L. Blumenschein G. Jr. Fayette J. Guigay J. Colevas A. D. Licitra L. (2016). Nivolumab for Recurrent Squamous-Cell Carcinoma of the Head and Neck. N. Engl. J. Med. 375 (19), 1856–1867. 10.1056/NEJMoa1602252 27718784 PMC5564292

[B15] Friedman C. F. Proverbs-Singh T. A. Postow M. A. (2016). Treatment of the immune-related adverse effects of immune checkpoint inhibitors: a review. JAMA Oncol. 2 (10), 1346–1353. 10.1001/jamaoncol.2016.1051 27367787

[B16] Gong J. Chehrazi-Raffle A. Reddi S. Salgia R. (2018). Development of PD-1 and PD-L1 inhibitors as a form of cancer immunotherapy: a comprehensive review of registration trials and future considerations. J. Immunother. Cancer 6 (1), 8. 10.1186/s40425-018-0316-z 29357948 PMC5778665

[B17] Haanen J. Carbonnel F. Robert C. Kerr K. M. Peters S. Larkin J. (2017). Management of toxicities from immunotherapy: ESMO Clinical Practice Guidelines for diagnosis, treatment and follow-up. Ann Oncol. 28 (suppl_4), iv119–iv142. 10.1093/annonc/mdx225 28881921

[B18] Herbst R. S. Baas P. Kim D. W. Felip E. Pérez-Gracia J. L. Han J. Y. (2016). Pembrolizumab versus docetaxel for previously treated, PD-L1-positive, advanced non-small-cell lung cancer (KEYNOTE-010): a randomised controlled trial. Lancet 387 (10027), 1540–1550. 10.1016/S0140-6736(15)01281-7 26712084

[B19] Higgins J. P. Altman D. G. Gotzsche P. C. Juni P. Moher D. Oxman A. D. (2011). The Cochrane Collaboration’s tool for assessing risk of bias in randomised trials. BMJ 343, d5928. 10.1136/bmj.d5928 22008217 PMC3196245

[B20] Higgins J. P. Thompson S. G. Deeks J. J. Altman D. G. (2003). Measuring inconsistency in meta-analyses. BMJ 327 (7414), 557–560. 10.1136/bmj.327.7414.557 12958120 PMC192859

[B21] Hodi F. S. O’Day S. J. Mcdermott D. F. Weber R. W. Sosman J. A. Haanen J. B. (2010). Improved survival with ipilimumab in patients with metastatic melanoma. N. Engl. J. Med. 363, 711–723. 10.1056/NEJMoa1003466 20525992 PMC3549297

[B22] Huang X. Lin J. Demner-Fushman D. (2006). Evaluation of PICO as a knowledge representation for clinical questions. AMIA Annu. Symp. Proc. 2006, 359. 10.1007/11878773_65 PMC1839740 17238363

[B23] Ji M. Liu Y. Li Q. Li X. Ning Z. Zhao W. (2016). PD-1/PD-L1 expression in non-small-cell lung cancer and its correlation with EGFR/KRAS mutations. Cancer Biol. Ther. 17 (4), 407–413. 10.1080/15384047.2016.1156256 26954523 PMC4910919

[B24] Jing W. Li M. Zhang Y. Teng F. Han A. Kong L. (2016). PD-1/PD-L1 blockades in non-small-cell lung cancer therapy. Onco Targets Ther. 9, 489–502. 10.2147/OTT.S94993 26889087 PMC4741366

[B25] Khan M. Lin J. Liao G. Tian Y. Liang Y. Li R. (2018). Comparative analysis of immune checkpoint inhibitors and chemotherapy in the treatment of advanced non-small cell lung cancer: a meta-analysis of randomized controlled trials. Medicine (Baltimore) 97 (33), e11936. 10.1097/MD.0000000000011936 30113497 PMC6113026

[B26] Konishi J. Yamazaki K. Azuma M. Kinoshita I. Dosaka-Akita H. Nishimura M. (2004). B7-H1 expression on non-small cell lung cancer cells and its relationship with tumor-infiltrating lymphocytes and their PD-1 expression. Clin. Cancer Res. 10 (15), 5094–5100. 10.1158/1078-0432.CCR-04-0428 15297412

[B27] Luo W. Wang Z. Tian P. Li W. (2018). Safety and tolerability of PD-1/PD-L1 inhibitors in the treatment of non-small cell lung cancer: a meta-analysis of randomized controlled trials. J. Cancer Res. Clin. Oncol. 144 (10), 1851–1859. 10.1007/s00432-018-2707-4 30019319 PMC11813414

[B28] Ma K. Lu Y. Jiang S. Tang J. Li X. Zhang Y. (2018). The relative risk and incidence of immune checkpoint inhibitors related pneumonitis in patients with advanced cancer: a meta-analysis. Front. Pharmacol. 9, 1430. 10.3389/fphar.2018.01430 30618738 PMC6297260

[B29] O’Kane G. M. Labbe C. Doherty M. K. Young K. Albaba H. Leighl N. B. (2017). Monitoring and management of immune-related adverse events associated with programmed cell death protein-1 axis inhibitors in lung cancer. Oncologist 22 (1), 70–80. 10.1634/theoncologist.2016-0164 27534573 PMC5313273

[B30] Philips G. K. Atkins M. (2015). Therapeutic uses of anti-PD-1 and anti-PD-L1 antibodies. Int. Immunol. 27 (1), 39–46. 10.1093/intimm/dxu095 25323844

[B31] Postow M. A. Sidlow R. Hellmann M. D. (2018). Immune-related adverse events associated with immune checkpoint blockade. N. Engl. J. Med. 378 (2), 158–168. 10.1056/NEJMra1703481 29320654

[B32] Puzanov I. Diab A. Abdallah K. Bingham C. O. 3rd Brogdon C. Dadu R. (2017). Managing toxicities associated with immune checkpoint inhibitors: consensus recommendations from the Society for Immunotherapy of Cancer (SITC) Toxicity Management Working Group. J. Immunother. Cancer 5 (1), 95. 10.1186/s40425-017-0300-z 29162153 PMC5697162

[B33] Reck M. Rodriguez-Abreu D. Robinson A. G. Hui R. Csoszi T. Fulop A. (2016). Pembrolizumab versus chemotherapy for PD-L1-positive non-small-cell lung cancer. N. Engl. J. Med. 375 (19), 1823–1833. 10.1056/NEJMoa1606774 27718847

[B34] Rizvi N. A. Hellmann M. D. Snyder A. Kvistborg P. Makarov V. Havel J. J. (2015). Cancer immunology. Mutational landscape determines sensitivity to PD-1 blockade in non-small cell lung cancer. Science 348 (6230), 124–128. 10.1126/science.aaa1348 25765070 PMC4993154

[B35] Robert C. Schachter J. Long G. V. Arance A. Grob J. J. Mortier L. (2015). Pembrolizumab versus ipilimumab in advanced melanoma. N. Engl. J. Med. 372 (26), 2521–2532. 10.1056/NEJMoa1503093 25891173

[B36] Sterne J. A. Gavaghan D. Egger M. (2000). Publication and related bias in meta-analysis: power of statistical tests and prevalence in the literature. J. Clin. Epidemiol. 53 (11), 1119–1129. 10.1016/S0895-4356(00)00242-0 11106885

[B37] Su Q. Zhang X. Shen X. Hou Y. Sun Z. Gao Z. H. (2018). Risk of immune-related colitis with PD-1/PD-L1 inhibitors vs chemotherapy in solid tumors: systems assessment. J Cancer 9 (9), 1614–1622. 10.7150/jca.24200 29760800 PMC5950591

[B38] Sznol M. Postow M. A. Davies M. J. Pavlick A. C. Plimack E. R. Shaheen M. (2017). Endocrine-related adverse events associated with immune checkpoint blockade and expert insights on their management. Cancer Treat. Rev. 58, 70–76. 10.1016/j.ctrv.2017.06.002 28689073

[B39] Tan S. Zhang H. Chai Y. Song H. Tong Z. Wang Q. (2017). An unexpected N-terminal loop in PD-1 dominates binding by nivolumab. Nat. Commun. 8, 14369. 10.1038/ncomms14369 28165004 PMC5303876

[B40] Wang Y. Zhou S. Yang F. Qi X. Wang X. Guan X. (2019). Treatment-related adverse events of PD-1 and PD-L1 inhibitors in clinical trials: a systematic review and meta-analysis. JAMA Oncol. 1; 5 (7), 1008–1019 10.1001/jamaoncol.2019.0393 31021376 PMC6487913

[B41] Weber J. S. D’Angelo S. P. Minor D. Hodi F. S. Gutzmer R. Neyns B. (2015). Nivolumab versus chemotherapy in patients with advanced melanoma who progressed after anti-CTLA-4 treatment (CheckMate 037): a randomised, controlled, open-label, phase 3 trial. Lancet Oncol. 16 (4), 375–384. 10.1016/S1470-2045(15)70076-8 25795410

[B42] Zak K. M. Grudnik P. Magiera K. Domling A. Dubin G. Holak T. A. (2017). Structural biology of the immune checkpoint receptor PD-1 and its ligands PD-L1/PD-L2. Structure 25 (8), 1163–1174. 10.1016/j.str.2017.06.011 28768162

